# Reminiscences of the Late Dr. Physick, and a Few of His Contemporaries

**Published:** 1856-12

**Authors:** 


From the N. J. Med. & Surgical Reporter.

### *Reminiscences of the Late Dr. Physick, and a few of his Contemporaries.*

To the Editor of the Reporter.

*Dear Sir:* I agree in the opinion expressed by you a short time since to the effect, that those biographies of great men, which are confined exclusively to details of their professional abilities and duties, are not always calculated fully to satisfy the closer and more intimate inquiries even of such students as may be the most eager to follow after them in their particular callings, and the best disposed to confide in them as practical guides and teachers of the sciences.

We can, all of us, admire and form either a higher or a lower estimate of men's acquirements as orators, statesmen, and divines, by witnessing their public displays, and of the skill of physicians and surgeons by reading their treatises and the records of their successful operations. But admire the exalted members of those several professions as we may, on account of what we hear and see of them in their public careers, there still remains a strong and natural desire in most minds to know something, however trivial, of their private habits, actions, and sometimes, as exhibited behind the scenes, and when they were protected from public scrutiny. We are still anxious to see them stripped, if possible, of all the formalities and technicalities of the schools; of their glowing sentences and commanding attitudes and gestures as orators; of their fervor and ingenuity as statesmen; of their solemnity as divines; of their mystery and penetrativeness as physicians.

But while we confess that, in common with most readers of biography, we should be pleased to find in the published lives of the generality of our great men some well-authenticated details of their private characters and social habits, we are at the same time ready to admit that in many cases such details would have no very beneficial effects upon private or public morals. Unfortunately, the old saying is too true—Virtue is not always allied with greatness.

The private lives of really good and great men are usually so simple, so unpretending, and, consequently, so free from "striking incidents," that it is not easy, even after much labor of research, to distinguish them from those of other virtuous men who have trod the paths of existence in obscurity; while on the other hand, the lives of many men who had become great and famous in the arts and sciences, could be crowded with details that would shock not only religion and morality, but the commonest feelings of our nature.

So truly were the life and character of the late Dr. PHILIP SYNG PHYSICK identified with all that is really "good and great," as included in the class first mentioned, that I already regret the promise I made to furnish you with some reminiscences of his domestic peculiarities; for, when I endeavor to look back to a period now distant more than half a century, I can remember but little that might not be comprised in the simple but comprehensive expression "really good and great."

As I informed you in conversation, I was between eleven and twelve years of age when I was first introduced to the eminent physician and surgeon whose name has just been mentioned, in the capacity of "traveling companion;" an expression which you will easily understand to allude to the little fellow that was formerly seen occupying the left hand corner of the "doctor's chair," as it stood at the door of some sick citizen, with the reins of the horses' bridle in one hand and a book in the other. I say as you might *formerly* have seen those things; for, although I often observe boys now in pretty much the same positions their predecessors occupied in my time, they are too generally minus the book. I hope the doctors, their employers, will attend to this, and, perhaps, their boys will remember them with tears of gratitude fifty years hence.

But I beg pardon, Mr. Editor, for this digression, as well as for this apparent egotism. I say apparent, because, as the travelling companion of the doctor, I shall not always be able to speak of him without, as I apprehend, making a reluctant but necessary allusion to your humble correspondent.

The interesting introduction referred to above took place on a cold morning early in the winter of 1803. There—that is to say, in his office, at his residence, the first door below Sixth in Arch Street—you might have seen the dignified Doctor Physick in familiar conversation with the poor mother of a poor little boy, for whom she was endeavoring to procure a comfortable home in exchange for very light services. And there you might also have seen three doctor's students peering over the tops of their books, evidently more intent upon listening to the terms of the "bargain and sale" than interested in the medical and surgical terms in the printed pages before them. When the treaty was concluded, I thought they looked pleased at me, for it stipulated, on the doctor's voluntary proposal, that I should be sent to Benjamin Tucker's Night School every winter until I was old enough to be put to a trade, or otherwise disposed of. The doctor then addressed me, and admonished me to be a good boy in a voice so silvery, so kind, and so touching to my feelings, that for the first time I ventured to look up into the face of so fine a gentleman, through my tears, and was met by one of the most benignant and animating smiles I ever received from the countenance of man. No person that I have ever met with since in the wide world, and I have watched the countenances of thousands in many countries, ever smiled such an expressive smile of benevolence as Dr. Physick. Its beams seemed radiant with goodness that came glowingly from the heart, and they could not but fall soothingly upon the young and unpractised hearts of those for whose consolation and encouragement they were often intended. But here I must correct myself. There was, indeed, one other person at whom I delighted to steal a glance while I lived with the doctor—a plainly dressed lady of most engaging mien, whose face would radiate, like the doctor's, with benevolence—whose voice and tones could touch, like his, even the heart of a friendless and apparently thoughtless boy. That lady was Dr. Physick's sister, the mother of the late Dr. Dorsey. How little do those great people know of the human heart who imagine that poverty is strong enough to shut out all the finer sensibilities of the heart, or deprive it of the secret pleasure it derives from the contemplation, and in silently venerating and loving the virtues, of those to whom custom forbids them to speak.

In his dress Dr. Physick was remarkably neat, and even particular, as, indeed, were all persons of his day, who were desirous of maintaining, in their own personal right, the distinctive appellation of gentleman. Yet his neatness and evident attention to dress were by no means singular, neither did they produce anything in his appearance or manners which resembled stiffness, formality, or frivolity. There were many well-dressed fops and dandies of the old school in his days, as there are of the new school in our days ; and the former had traits which distinguished them from true gentlemen, just as individuals of the same *genus homo* present peculiarities to us now.

The dress of Dr. Physick was, therefore, such as was considered essentially necessary to the dignity of a professional man. Since his time, however, great changes have taken place in the costumes, and many changes also in the manners of men. The morning toilet of a practising physician, as may easily be judged, was an important and a troublesome affair some fifty or sixty years ago. Few medical men in this era of hurry and excitement take time to shave off their beards, and none of them would be so perfectly silly and ridiculous as to powder their hair !

It was the common practice of Dr. Physic to rise early in the morning, as it was to retire early to rest. The first duty he performed was to read a chapter in the New Testament, a copy of which was deposited in the recess of the back parlor window at which he shaved himself.

This daily practice, let me here remark, did not escape my attention, young and uneducated as I was. I had, indeed, read portions of the Holy Scriptures in a class of boys at school, and heard them read by my mother, but I had attached no definite importance to their character or meaning. Observing, however, how quietly and how reverentially the doctor read his lesson every morning, I conceived a new idea in regard to the intrinsic worth and sacredness of the volume ; which idea, perhaps, has ever since retained, at least, a portion of its early influence over all my subsequent reflections on religious questions, especially when disposed to doubt or reject. So much, then, in brief, for the good examples set by great men, and for the deep and lasting impressions they make on the susceptible minds of youth.

When fully prepared to visit his patients, no sample of the physician or of any other profession of the present day could well be compared to Dr. Physick for dignity and gracefulness of appearance. He generally wore a gray or light-colored dresscoat, cut after a fashion which is not yet entirely out of date ; a white or neatly figured double-breasted Marseilles vest, light cassimere small clothes, white silk stockings, shoes or boots, according to the season, highly polished One peculiarity of his dress was a fine muslin cravat, the corners of which were tied in front, forming a knot very much resembling the intersecting leaves of a full-blown rose. And thus tastefully attired, but in all which, as we have before remarked, there was not the least show of affectation or foppishness, and with a cheerful and happy countenance, he would take his seat in "the chair" by the side of his traveling companion, seize the reins, give a chirrup to the quiet old mare, and sally forth upon his daily rounds through the city and country, carrying hope and consolation, if not health and life, by his very presence into the gloomy chambers of his patients.

But in attempting thus minutely to describe the outward characteristics of Dr. Physick, let it not be understood that he was the only one of the medical profession in his day who was the "observed of all observers," or that his celebrated contemporaries were less careful of their dignity and personal appearance as gentlemen than he was. Often, indeed, does an active memory, which warmly clings to the "light of other days," gratify me with a backward journey to the men and the fashions of my youth. Often, as I pass through some of the old and once familiar quarters of the city now hemmed in or driven from their ancient proprieties by the bustle and confusion of business and commerce, does my imagination re-assemble at the door of some quaint family mansion of the olden time—now converted, perhaps, into a rum distillery or a low tavern—the forms of Dr. Rush, Dr. Wistar, and Dr. Physick, standing, as I have seen them stand, apparently conversing on familiar subjects at the close of an important consultation in the case of some prominent actor in the war of the Revolution, whose name has long since been forgotten, notwithstanding the military fired three rounds over his grave, and the sweet-toned bells of Christ Church tolled his requiem in melancholy numbers.

In such reveries as this, Mr. Editor, which are by no means unpleasant at my time of life, I examine once more, with all the intensity of my boyish curiosity, the marked faces, and all the outward peculiarities of the three great medical counsellors, whose honored names have just been mentioned; their powdered heads, their striking attitudes, their breeches, their knee-buckles, their silk stockings, their black and shining shoes, and above all, the ease and dignity which so greatly distinguished all three from those who passed them by, but not without observation and the occasional interchange of such bows as we seldom see in these days of practical heedlessness. There I behold once more the mild, the thoughtful, the penetrating eyes and attenuated features, the compressed lips, the folded arms, and slender form of the signer of the Declaration of Independence. There, also, I see again the stouter and more compact figure, the ruddier face full of health and animation, the hands clenching each other behind, of the second on my list of old acquaintances, made such only by my having frequently seen them. And there, again, I see the more erect form, the symmetrically proportioned limbs, but, above all, the benign and manly look, so full of truthfulness and pure elevation of purpose, the face at once so classical in its contour, and so firm in all its lineaments of my respected and admired patron, Dr. Physick.

How vivid must have been the impress made upon the minds of the older and keener observers and admirers of those great lights of the medical profession, seeing that the personal appearance of each is still retained with accuracy in the mind of one who has seen but little of either of them since the year 1806!

After his morning visits, which included his attendance at the Pennsylvania Hospital, and, if there was yellow fever at the Wigwam, on the Schuylkill, or, perhaps, at Bush Hill, Dr. Physick returned home and dined with his family on the most simple fare, indulging in a few luxuries, but in no excess. Indeed, he had no time to spare for anything of that nature, his afternoons being devoted to his lectures, which were then delivered in the Pennsylvania Hospital; or, in the summer season, to his patients in the country and suburbs of the city. On the latter occasions, when we were clear of the rough pavements, pursuing our way upon some quiet road, or through a shaded lane, such as in those days led in every direction from the city—they are crowded streets now—my humble but susceptible heart was often delighted and encouraged to hope for something in the future; from the doctor's condescension towards me, his familiarity in asking me questions relating to the progress I had made at school, encouraging me to read, even calling me his little student, and telling me what he proposed to do for me when the proper time should come, if I continued to be a good boy. Alas! that time never came! But that it did not come was no fault of the good doctor's, and I may say, also, that it was not *entirely* mine.

On occasions like those mentioned, Dr. Physick would often sing, in low and sweet tones, snatches from such songs as the "Beggar Girl," "Far Beyond the Mountains," "Banks of the Dee, and other simple and popular airs of that day. The tranquillity of the scenes through which we were passing seemed to have a genial influence on his musings and reflections, creating feelings of such exquisite pleasure as could only be expressed, as expressed they must be, in words of hope and kindness to the poor boy that sat beside him, or in the touching notes of a favorite ballad. He had, indeed, just left the presence of his own dear children, for whom he always manifested the most tender affection, and, young as I was, I thought *that* was one of the reasons for his gentleness and sympathy towards me, who had no claims but those of humanity upon his attention. But he did not know how deeply my young heart was affected by his looks, by his words, and by his most trivial actions; how it was silently receiving memorials and impressions that were never to be effaced so long as life endured.

To convince you, Mr. Editor, how intimately real simplicity and goodness of heart were blended with true greatness in the character of Dr. Physick, permit me here to relate an incident, which, artless as it may seem, will speak volumes for the generous susceptibility of his nature. In his family there lived a pleasant and lively girl, about my own age, who was employed as an under nurse, or attendant on the Doctor's two daughters, Sarah and Susan. During the summer, the family, or the greater portion of it, removed to the country, and occupied an old-fashioned mansion which stood on the brow of a hill which overlooked the Schuylkill, immediately in the rear of what was then called the Upper Ferry—Fairmount now. The house was an ancient affair. It had been used, in revolutionary times, as a hospital and barracks for the British, when in occupation of Philadelphia. There were some evidences of this fact remaining, especially in the cuts and gashes which were to be seen in the oaken floors, on which it was supposed the soldiers had been in the practice of chopping and splitting their fire-wood. The old "country-seat" had the bad but common reputation of nearly all old houses, even in that advanced era of intelligence, of being "haunted." In the middle of an extremely warm and oppressive night, near the close of the summer of 1804, the whole household was awakened by a loud crash, which echoed through every apartment, and which made a noise as if the entire contents of a corner closet—glass doors, china-ware, crockery, silver-ware, and all—had been suddenly broken to atoms. I was sleeping on a cot on the landing at the head of the garret stairs. I awoke in great terror, and although the light of a brilliant moon shown in at the window, and although the heat was intolerable, I wound myself, head and all, so tightly in the bedclothes, that I would in all probability have smothered had not the Doctor come to my relief. When my sight and free respiration were restored, there he stood, with a war-like weapon in one hand, and a lighted candle in the other, buttoned up in his flannel wrapper, and flanked on either side by the coachman and waiter, and covered in the rear by the housekeeper and some half a dozen other females, comprising the cook, nurses, chambermaids, etc. Their object was, of course, to discover the cause of the noise which had so dreadfully disturbed the house, every other department of which had been examined in vain, including the fearfnl parlor in which the confusion had seemed to commence. But there stood the closet in its usual corner, silent and undisturbed; and opposite, and equally unconscious, the tall eight-day clock was swinging its pendulum, and clicking away the solemn hour of midnight. And next they thought of mounting up to the garret, to ascertain what John, who about that time was said to be growing somewhat mischievous, had to do with making the racket. But it was to no purpoe that I was stripped of my covering, for which I then had other use than to screen me from the ghost. In vain was I directed to make various movements and contortions of my light and slender body. Nothing could be adduced, from all the changes of position I was able to produce, that it was "I that did it," and I was finally left to my reflections, but not to repose.

In the morning, after the strange proceedings of the previous night had been thoroughly examined and discussed by the servants, under the monitorship of the venerable housekeeper, I met the little nurse with the children on the shaded lown in front of the old mansion. "Oh, John," she said to me, "I am sure something dreadful is going to happen. That noise we heard last night is a death-warning for some one, but for whom we cannot tell. It may be for *me*, it may be for *you*, but it is for some one here, for I heard my aunt say that such noises foretold the death of my dear mother and father."

Had I been a young philosopher of the present enlightened age, I would have boldly combatted the superstitious idea which had taken possession of the poor girl's mind. But, unfortunately, I was as susceptible as herself to ghostly apprehensions, and could only "wonder who it would be." I had read and studied and sung many of the old ballads, such as the "Ghost that came to Margaret's door," and was not prepared to ridicule or to deny the opinions advanced by the simple-minded girl.

When the Doctor got into the chair to drive to the city, I thought that he did not look as pleasant and cheerful as usual. His countenance wore a disturbed and melancholy cast, not unlike that which I had remarked in the looks of the little nurse. Perhaps *he* wondered, as *we* had done, "who it would be." So I thought then.

In the evening (having dined, as was his custom, at his house in the city), the Doctor returned to his family seat. As we approached the mansion, I was surprised not to see my gentle friend, the orphan nurse, on the lawn, in company with the children, where I had been in the habit of meeting with her when we arrived, and chatting a few moments about what was going on in that portion of the family left in charge in the city. She was not there. She had not hidden behind any of the great oak-trees, which, in her playfulness, she was sometimes wont to do. The children met their father alone. I felt alone, sad, and disappointed, and, I think, apprehensive, for I was still *wondering who it would be.*

"Where is Kitty?" asked the Doctor, in a kind tone, apparently as much surprised and as anxions at her absence as I was. "Poor Kitty is sick," answered Sarah, the eldest of the children. "Kitty is, indeed, very ill," said Mrs. Physick, as she greeted the Doctor.

Of course, the good Doctor lost no time in visiting the sick girl. He found her, as I was told by one who attended on her, in a "raging fever, and completely out of her head." Of course he did everything that his skill and his benevolence could suggest to alleviate her sufferings. But her too sensitive mind had received a shock greater than her delicate nervous system could sustain, and she sank rapidly. Such was her excitability, that, on repeated applications, I was forbidden to see her; but, in despite of the interdiction, on the fourth evening of her illness, I stole noiselessly up to her chamber. Here I found the Doctor standing at a window, with his back towards the open door at which I entered, conversing in low tones with the nurse; he did not at first observe me. Not so the patient. Her restless eye flashed upon me in an instant, and she exclaimed, in a wild but tender voice, "John, John, you need wonder no longer who that terribie warning was meant for; it was for me—for *me*, and not for *you*." Hearing her words, the Doctor started, and seeing me approaching the bed of the dying girl, mildly but firmly rebuked me for my intrusion, and bade me leave the room immediately. The intense light of her dark, expressive eyes followed me to the door, but they were soon after closed in the dim shadows of death. * * *

It was on a beautiful evening in September, and at an hour when the sun had so far gone down in the west as to cast a melencholy shade over the trees and shrubbery that bounded the deep, rocky, and narrow road that led up to the old "country-seat," that we arrived at a point at the foot of the hill, on our return from the city. Just as we reached it, we heard the rumbling and crashing among the rocks of a descending carriage, which, when it came fully in sight, proved to be an old-fashioned Jersey wagon, belonging to the Doctor, from the open front of which protruded the foot of the coffin which embraced the remains of the beloved and lovely little nurse. The chair was immediately drawn up on one side of the narrow road, to "let the coffin pass." "Poor Kitty!" exclaimed the Doctor, in an affectionate and mournful voice; "dear little girl! we shall never again meet her innocent face among the happy children." The coffin passed on its way to the house of Kitty's credulous aunt, from which the funeral was to take place; but the tender words of the Doctor were buried in my heart. When I looked up, the good man was brushing away the tear-drops from his cheeks, which, as they fell, were mingled with those of his humble companion.

But all such relations as the above, of such a man as was Dr. Physick, some of your readers will probably say, are puerile! Be it so. If such are not evidences of true greatness, then there has never been any true greatness exhibited to the world. But Dr. Physick was indeed a great man, a humane and a benevolent man, a Christian man, a brave, and a courageous man—all which he established in an eminent degree by his tenderness, his sympathy and his charity for the poor.

Still, it must not be supposed that the Doctor was entirely free from the infirmities which belong to our common humanity. He had his moments of irritability, if not passion, as well as other men; though he probably took more care than is usual to restrain his natural impulses. I have witnessed those little gusts of feeling, however, which will sometimes darken the most placid brows, and was myself, no doubt, often the occasion of them, for I was only a wayward boy at best. The least manifestation of disobedience, impertinence, or of neglect of my person or dress, was rebuked at once by a single glance of displeasure, which was not to be misunderstood or forgotten, even by a person of my years. It was thus with his students, some of whom were, as I presume students are at this day, given somewhat to fun and frolic. The dignity which was ever a conspicuous trait in the character of their preceptor rendered it impossible for them to be anything but the most perfect gentlemen in his presence. I was formerly in the habit of meeting with several of those students, long after I had grown out of their recollection, and I was always forcibly reminded by their easy and courteous manners, even on the streets, of the personal character of Dr. Physick.

The last time I ever saw Dr. Physick, was some thirty-five years ago. I called upon him with a message from one of his poor patients. He was in his office, in rear of his residence, then in Fourth below Spruce Street, surrounded by a crowd of sick and diseased persons of the same class, to whom he was busily engaged in administering, gratis, all the alleviation his well-stored mind and generous heart could afford them. He rests in peace, for it was his object to live in peace and charity with all mankind.

Very respectfully yours.

Burlington, N. J., Oct. 1856.

